# Traditional Chinese Medicine Constitution Discrimination Model Based on Metabolomics and Random Forest Decision Tree Algorithm

**DOI:** 10.1155/2022/3490130

**Published:** 2022-05-16

**Authors:** Chaodong Huang, Yufeng Chen, Bingtao Li, Qiyun Zhang, Li Jiang, Bin Nie, Lihua Chen, Hui Jian, Guoliang Xu

**Affiliations:** ^1^Research Center of Differentiation and Development of Basic Theory of TCM, Jiangxi University of Chinese Medicine, Nanchang, China; ^2^School of Computer Science, Jiangxi University of Chinese Medicine, Nanchang, China; ^3^Jiangxi Provincial Key Laboratory of Etiological Biology of Traditional Chinese Medicine, Nanchang, China; ^4^Jiangxi University of Chinese Medicine Key Laboratory of Modern Preparation of Traditional Chinese Medicine, Ministry of Education, Nanchang, China; ^5^School of Basic Medicine, Jiangxi University of Chinese Medicine, Nanchang, China; ^6^Jiangxi Provincial Key Laboratory of Traditional Chinese Medicine Pharmacology, Nanchang, China

## Abstract

Constitution refers to the comprehensive and relatively stable characteristics of the genetic or acquired morphological structure, physiological function, and psychological state in the process of human individual life. A special metabolomics data processing method is established to find the unique *m/z* value of each constitution. Combined with the random forest decision tree algorithm, the discrimination model of 9 constitutions in traditional Chinese medicine is constructed, and the model is verified and tested. The test results show that the classification accuracy of each constitution is higher than 80%, indicating that the model can well identify nine constitutions of traditional Chinese medicine. The classification accuracy is related to the difficulty of distinguishing between constitutions. In a word, this study provides a fast and accurate method to distinguish the constitution of traditional Chinese medicine, provides an objective representation for the classification and judgment of clinical constitution of traditional Chinese medicine, and provides a scientific basis for the modernization of traditional Chinese medicine.

## 1. Introduction

Constitution refers to the comprehensive and relatively stable characteristics of human individual's morphological structure, physiological function, and psychological state formed by heredity or acquired during human individual's life. Under the physiological state, the difference of constitution is reflected in the difference in response and adaptability to external stimuli, as well as the susceptibility to some pathogenic factors and the tendency of disease development. The study of constitution is helpful to analyze the relationship between different constitution types and diseases and provides a basis for the prevention and treatment of diseases [1].

“TCM constitution Classification Criteria” and “TCM Constitution Classification Scale of 9 Basic Constitutions” developed by Professor Wang Qi of Beijing University of Chinese Medicine are currently recognized as TCM constitution diagnostic criteria [2]. The constitution can be divided into 9 kinds, namely, Pinghe constitution, Qixu constitution, Yangxu constitution, Yinxu constitution, Tanshi constitution, Shire constitution, Qiyu constitution, Xueyu constitution, and Tebing constitution, among which Pinghe constitution is a relatively healthy constitution and the rest of them are biased constitution [3].

Pinghe (peaceful) constitution [4–6]: a strong physical state, which is characterized by moderate posture, ruddy complexion, and energetic state; Qixu (qi deficiency) constitution: a physical condition characterized by weak breath and low function of the body and viscera; Tanshi (phlegm dampness) constitution: it is a physical state characterized by stagnation in water and fluid and condensation of phlegm and dampness, with viscosity and turbidity as the main characteristics; Shire (damp heat) constitution: a physical condition characterized by damp heat; Yangxu (Yang deficiency) constitution: a physical condition characterized by deficiency of Yang qi and deficiency of cold; Qiyu (qi depression) constitution: a physical condition characterized by introversion, instability, melancholy, fragility, sensitivity, and paranoia due to long-term emotional stagnation and qi stagnation; Yinxu (Yin deficiency) constitution: due to the deficiency of Yin fluid such as body fluid, essence, and blood in the body, the physical state is characterized by Yin deficiency and internal heat; Xueyu (blood stasis) constitution: it refers to the physical condition that there is a potential tendency of poor blood operation or the pathological basis of blood stasis internal resistance in the body and shows a series of external signs; Tebing (special) constitution: manifested as a specific constitution, mostly refers to a physical defect caused by congenital and genetic factors, including congenital and genetic physiological defects, congenital and genetic diseases, allergic reactions, and primary immune defects.

Compared with the highly subjective TCM syndrome diagnosis criteria and syndrome differentiation index system, metabolomics shows the overall changes of metabolic products in the body and predicts the internal changes of the body through the whole. Its characteristics of integrity are consistent with the thinking mode of “holistic view,” “syndrome differentiation and treatment,” and “outside the department, inside the department” in TCM [7]. Through the dynamic tracking analysis of metabolites in the metabolic cycle, with the help of multivariate statistical analysis method, it can reflect the changes of functional status of organisms caused by internal and external factors such as genes and environment instantly and sensitively, providing objective information for clinical practice. Compared with other omics, metabolomics is closer to phenotype and has similarities with TCM constitution identification [8–10]. Random forest (RF) algorithm is an ensemble learning method. On the premise of taking CART decision tree as the base classifier, RF introduces random selection samples and random selection features in the training process of decision tree to form multiple decision trees. The final classification result is the mode or average value of output categories of all decision trees in the forest. RF has the advantages of high prediction classification accuracy, strong generalization, and fast training speed so that it can be used for classification, regression, and feature importance analysis. Therefore, we combine metabolomics with algorithm to make a preliminary analysis of TCM constitution from the “scientific” and “objective” perspective, which will bring a more comprehensive understanding to reveal the phenomenon of TCM constitution [11–13].

By recruiting volunteers of 9 kinds of constitution of TCM, this study uses the UPLC-Q-TOF-MS technique to detect and collect the serum of all volunteers so as to establish the discriminant model of 9 kinds of constitution of TCM through the RF algorithm, which provides a new method and idea for the study of physical classification and provides the objective basis for revealing the principle of theoretical physical classification of traditional Chinese medicine.

## 2. Materials and Methods

### 2.1. The General Information

#### 2.1.1. Volunteer Recruitment and Ethics Lot Number

This study recruited ordinary people in two centers (Jiangxi University of Chinese Medicine and Physical Examination Department of Affiliated Hospital of Jiangxi University of Chinese Medicine) through oral publicity, recruitment posters, and public account publicity [14]. The volunteers who met the inclusion criteria knew the details of the experiment and signed the informed consent to complete the inclusion. The batch number of ethics is JZFYLL20200914015.

#### 2.1.2. Constitution Diagnostic Criteria

The constitution identification system of tongue and pulse information acquisition of DS01-A was adopted, combined with the standard of ZYYXH/T157-2009 (Classification and Determination of TCM Constitution) issued by China Association of Traditional Chinese Medicine, and the Table of Classification and Determination of TCM Constitution in the standard was filled in. The criteria for determination of mild constitution and 8 kinds of biased constitutions are shown in Table 1.

#### 2.1.3. Inclusion Criteria

1. Meet the classification criteria of TCM constitution
2. General population aged ≥18 years old
3. Voluntarily participate and sign informed consent

#### 2.1.4. Exclusion Criteria

1. People with mixed constitution
2. Pregnant or lactating women
3. Those with mental, cognitive, conscious disorders who cannot cooperate to complete the test
4. Patients with serious primary diseases of heart, brain, and hematopoietic system, serious liver and kidney damage, lung infection, malignant tumor, and mental diseases
5. Have participated in other research studies

#### 2.1.5. Inclusion-Related Examination

1. Complete the constitution test
2. Fill in the volunteer's personal information
3. Complete physical examination: height, weight, and blood pressure
4. Complete routine laboratory examinations: blood routine, urine routine, stool routine, electrocardiogram, liver function, kidney function, and coagulation.

### 2.2. Materials and Methods

#### 2.2.1. Method of Constitution Determination

Fill in all the questions in the “TCM Constitution Classification and Judgment Table,” score each question according to 5 grades, calculate the original score and transformation score, and determine the constitution type according to the standard.

(1) $$\begin{array}{c} \text{original score}=\text{add the score values of each entry},\\ \text{conversion score}=\left[\frac{\text{original score}-\text{number of items}}{\text{number of items}\times 4}\right]\times 100. \end{array}$$

#### 2.2.2. Reagent

Methanol, purchased from Merck (Germany), acetonitrile, purchased from Merck (Germany), formic acid from DikmaPure Company (USA), purchased from DikmaPure Company, (USA), leucine enkephalin from Waters Company (USA).

#### 2.2.3. Instrument

DS01-A Daosheng Four Diagnostic instrument (Daosheng, China), Waters Acquity UPLC Liquid Chromatography System, Q-TOF SYNAPT G2 HDMS (Waters Corporation, USA), high-speed refrigeration centrifuge (Termo Scientific, Germany), SpeedVac®SPD131Centrifuge enrichment system (Termo Scientific, Germany), VORTEX GENIUS 3 VORTEX oscillator (IKA, Germany), and Milli-Q Advantage A10 Ultra-pure Water Purifier (Milli-pore, USA).

#### 2.2.4. Sample Collection

The volunteers did not take any drugs before blood collection on the specified date of sample collection (after 10pm before blood collection, no food was taken). After blood collection, the volunteers stood for 3 h and centrifuged for supernatant, which was stored at −80°C after collection.

#### 2.2.5. Sample Preparation

Frozen serum samples were thawed at room temperature. Then, 100 *μ*L of the sample was placed in centrifuge tubes, and 300 *μ*L of methanol was added. The tubes were vortexed for 1 min, incubated for 3 h at 4°C, and then centrifuged (21300 × *g*, 10 min, 4°C). The supernatants were collected and dried by SpeedVac®, and the residues were reconstituted in 200 *μ*L of methanol: water (15 : 85). Then, the samples were vortexed for 1 min and centrifuged (30000 × *g*, 15 min, 4°C). The supernatants were collected and subsequently analyzed following a previously described UPLC-Q-TOF-MS-based untargeted metabolic profiling strategy [15–17].

#### 2.2.6. Methods for UPLC-Q-TOF-MS

*(1). UPLC Conditions.* Chromatographic column: Waters ACQUITY UPLC BEH C_18_ column (2.1 mm × 100 mm, 1.7 *μ*m) was used for chromatographic determination on A Waters ACQUITY UPLC BEH C_18_ column (2.1 mm × 100 mm, 1.7 *μ*m). The column temperature was 40°C, sample chamber temperature was 10°C, flow rate was 0.3 mL·min^−1^, sample size was 3 *μ*L, mobile phase A was 0.1% formic acid water, mobile phase B was acetonitrile, gradient elution conditions: 0∼2 min, 99% A; 2∼8 min, 99%∼60% A; 8∼16 min, 60%∼26%; 16∼26 min, 26%∼2% A; 26∼28 min, 2% A; 28∼28.1 min, 2%∼99% A; and 28.1∼32 min, 99% A [18].

*(2). Q-TOF-MS Conditions.* The ionization source temperature is 120°C. The flow rate of cone-hole gas is 50 L·h^−1^. The temperature of solvent removal was 400°C, and the flow rate was 800 L·h^−1^. In positive and negative ion modes, the capillary voltage was 3.0 kV, 2.5 kV, and the taper hole voltage was 40 V. In the positive mode, the extraction taper hole voltage was 80 V. The compensating voltage is 80 V in the negative ion mode. The mass number ranges from 50 to 1000 Da. To ensure the classification accuracy and repeatability of quality, sodium formate standard was used to establish the quality axis standard curve, and leucine enkephalin was used for real-time quality correction. The tandem mass spectrometry collision gas is argon, with low impact energy of 4 eV and high impact energy of 20–40 eV. In this experiment, the stability of quality control samples (QC) was investigated to ensure the stability of instrument detection. It is necessary to run five QC samples before sampling, so that the instrument can reach a stable state. And, a QC sample balance system is needed for 8 samples per walk [19–22].

#### 2.2.7. Instrument Stability Investigation and Data Processing

Pearson analysis was performed on 6 QC samples obtained in positive and negative modes, respectively, and correlation coefficients were calculated to investigate the stability of instrument detection.

#### 2.2.8. Data Processing

After importing the data into Progenesis QI 2.0 software (Waters Corporation, USA), a text file with retention time and mass-to-charge ratio information is obtained [23]. Variable quality control: based on the QC group samples, variables with a coefficient of variation greater than 30% in the QC samples were deleted. The preprocessed data matrix was imported into GraphPad Prism 8.0 software (GraphPad Software,USA), and the eight biased constitutions were compared with the Pinghe constitutions, respectively. The *m/z* values of the significant differences between the eight biased constitutions and Pinghe constitutions were obtained. The common *m/z* values of the eight biased constitutions differing from the Pinghe constitutions were found through the intersection method, and the common *m/z* values were removed to obtain the unique *m/z* values of the eight biased constitutions. This step was completed on the jvenn website (https://jvenn.toulouse.inra.fr/app/example.html), and the Venn diagram could be more intuitive.

#### 2.2.9. Building Discriminant Model

*(1). The Discriminant Model of 9 Kinds of Constitution of TCM Was Established by RF Algorithm* [24–26]. The unique *m/z* values of the eight biased constitutions obtained in 2.2.8 were used to establish the model, and the missing values in the data set were filled with the corresponding feature (*m*/*z*) mean values of each constitution. *n* training sets are obtained by random sampling of original training sets with replacement. *m* features are randomly selected for each training set to obtain *n* classification models, and then the optimal classification is determined by voting.

*(2). The 10-Fold Cross Validation Method Was Used to Test the Model.* 10-fold cross-validation refers to dividing the data into 10 equal pieces. Each time the data is classified, one of the pieces is selected as the test set and the remaining 9 pieces are selected as the training set. Repeat 10 times so that each piece of data is used for one test set and nine training sets. The advantage of this method is that, as much data as possible is used as training set data, and each training set data and test set data are independent of each other and completely cover the whole data set.

Classification accuracy is the probability that samples are correctly classified, and the calculation formula is as follows:

(2) $$\begin{array}{c} \text{classification accuracy}=\text{TP}+\frac{\text{TN}}{P}+N. \end{array}$$

TP (true positive): indicates the number of positive cases that are correctly predicted. TN (true negative): indicates the number of negative cases correctly predicted. *P* (positive sample): indicates the number of positive samples. *N* (negative sample): indicates the number of negative samples.

## 3. Results

### 3.1. Comparison of Clinical General Data

The results of clinical examination showed that all the 9 groups of volunteers met the requirements for inclusion, and there were no differences in other indicators except physical differences. In the Qixu group, there were 12 males and 37 females, with a mean age of 26.41 years (standard deviation 4.11). In the Tanshi group, there were 16 males and 33 females, with a mean age of 28.98 years (standard deviation 4.41). In the Pinghe group, there were 12 males and 37 females, with a mean age of 26.96 years (standard deviation 4.37). In the Shire group, there were 16 males and 33 females, with a mean age of 26.73 years (standard deviation 2.94). In the Yangxu group, there were 10 males and 39 females, with a mean age of 27.57 years (standard deviation 4.33). In the Qiyu group, there were 13 males and 36 females, with a mean age of 26.69 years (standard deviation 2.59). In the Yinxu group, there were 14 males and 35 females, with a mean age of 26.31 years (standard deviation 4.75). In the Xueyu group, there were 15 males and 34 females, with a mean age of 26.67 years (standard deviation 2.14). In the Tebing group, there were 11 males and 38 females, with a mean age of 28.51 years (standard deviation 4.21). There were no significant differences in the distribution of sex and age among the nine groups of constitutions, as analyzed by the chi-square test and analysis of variance, respectively (Table 2).

### 3.2. Metabolic Map of 9 Constitutions in TCM

**Figure 1:**
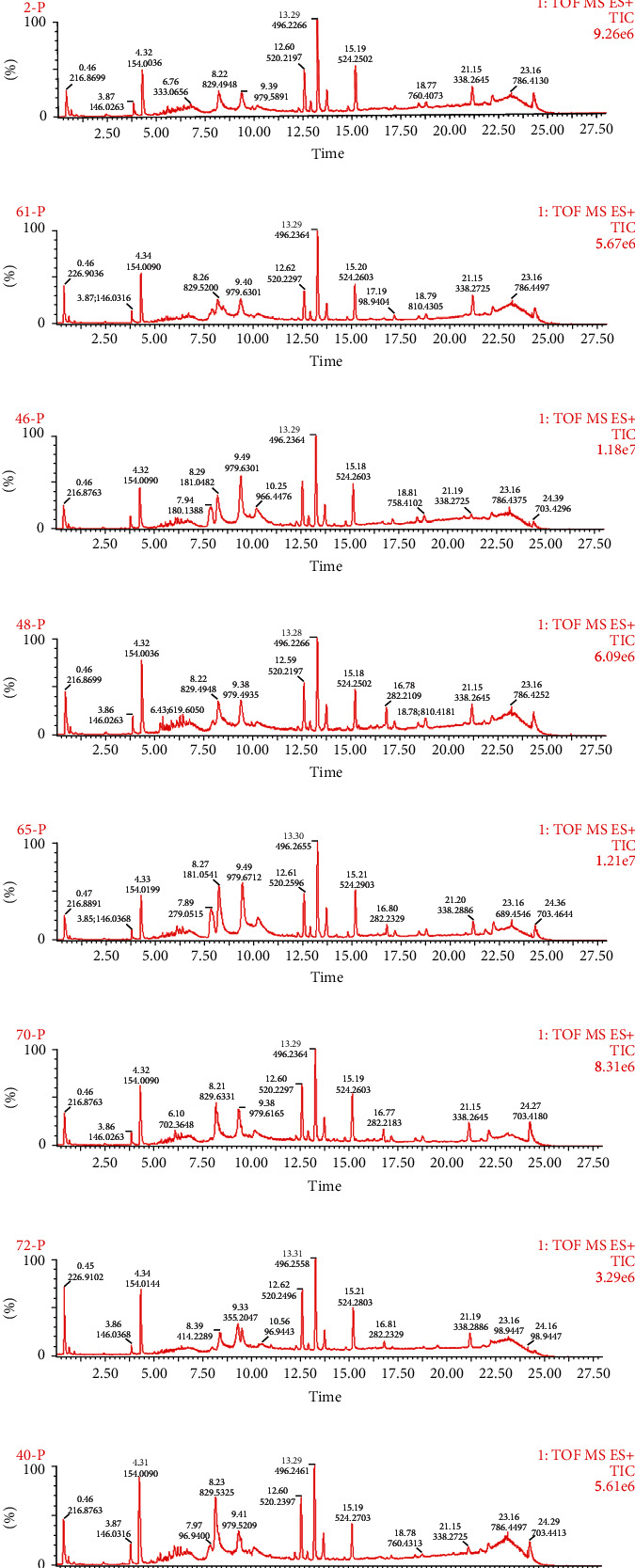
Total ion flow diagram of serum samples of 9 TCM constitutions under positive ion mode scanning. (a) Qixu group; (b) Tanshi group; (c) Pinghe group; (d) Shire group; (e) Yangxu group; (f) Qiyu group; (g) Yinxu group; (h) Xueyu group; (i) Tebing group.

**Figure 2:**
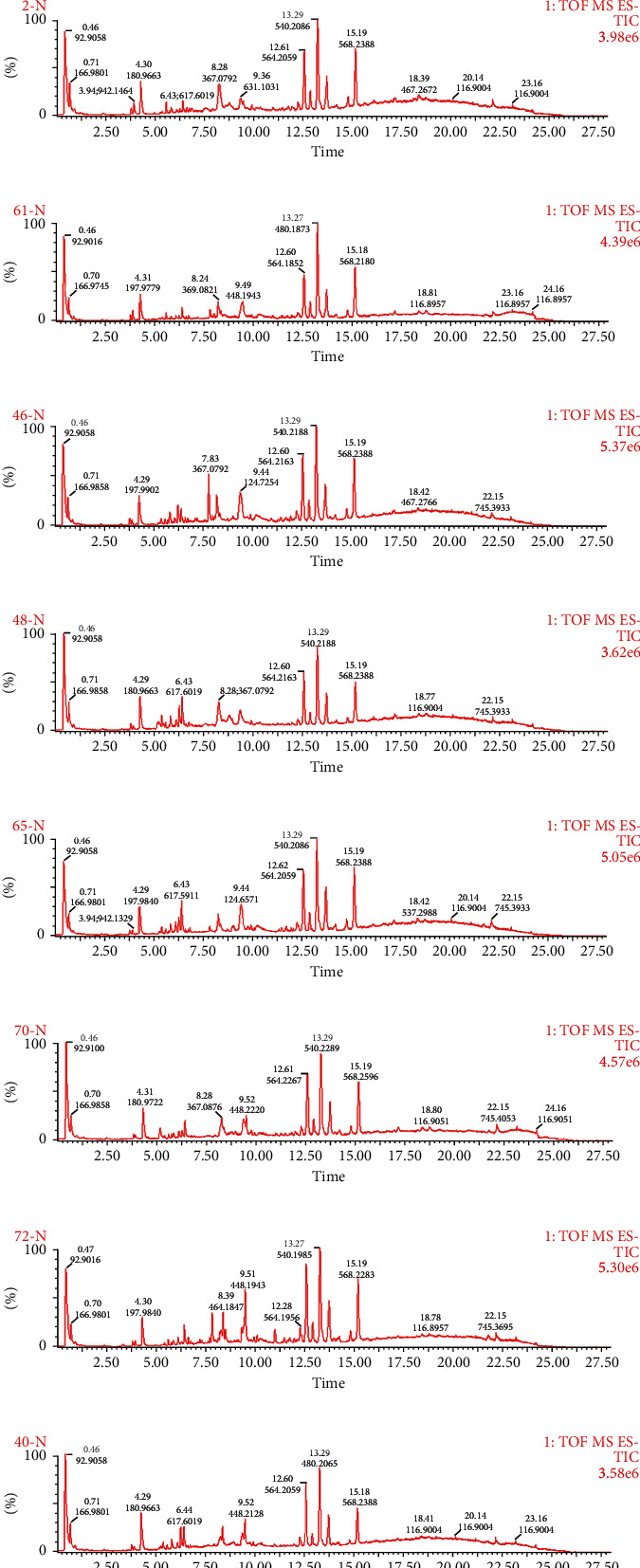
Total ion flow diagram of serum samples of 9 TCM constitutions under negative ion mode scanning. (a) Qixu group; (b) Tanshi group; (c) Pinghe group; (d) Shire group; (e) Yangxu group; (f) Qiyu group; (g) Yinxu group; (h) Xueyu group; (i) Tebing group.

Figures 1 and 2 (uploaded separately) show the total ion flow (TIC) of 9 TCM constitutions in positive and negative ion modes, respectively.

### 3.3. Instrument Stability Investigation

**Figure 3:**
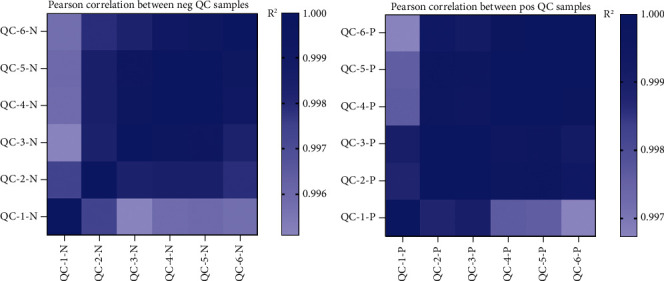
Pearson correlation analysis heat map. (a) Positive ion mode. (b) Negative ion mode.

By calculating Pearson correlation coefficient between 6 QC samples, the closer the correlation coefficient is to 1, the better the system stability is and the higher the data quality is. Correlation of QC samples is shown in Figure 3. As shown in the figure, *R*^2^ is greater than 0.99 and close to 1 in both positive and negative modes, indicating that the system has good stability and high data quality in the detection process.

### 3.4. *m/z* Values of the Eight Biased Constitutions Were Different from Those of Pinghe Constitutions

The results are shown in Table 3. The *m/z* values of the eight biased constitutions are obtained by comparing them with the Pinghe constitutions.

### 3.5. *m/z* Values Unique to Eight Biased Constitutions

**Figure 4:**
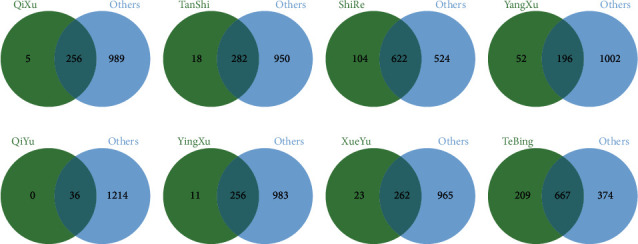
Venn diagram of one biased constitution and other biased constitutions under positive mode condition. (a) Qixu group; (b) Tanshi group; (c) Yangxu group; (d) Shire group; (e) Qiyu group; (f) Yinxu group; (g) Xueyu group; (h) Tebing group.

**Figure 5:**
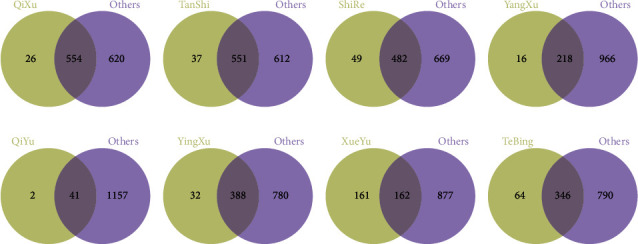
Venn diagram of one biased constitution and other biased constitutions under negative mode condition. (a) Qixu group; (b) Tanshi group; (c) Yangxu group; (d) Shire group; (e) Qiyu group; (f) Yinxu group; (g) Xueyu group; (h) Tebing group.

The intersection of one of the biased constitutions and the other seven biased constitutions was carried out to obtain the common *m/z* values of the eight biased constitutions. After removing the common *m/z* values, the unique *m/z* values of the eight biased constitutions were obtained, as shown in Figures 4 and 5.  Table 4 shows the number of unique *m/z* values of the eight biased constitutions.

### 3.6. The Test Results

**Figure 6:**
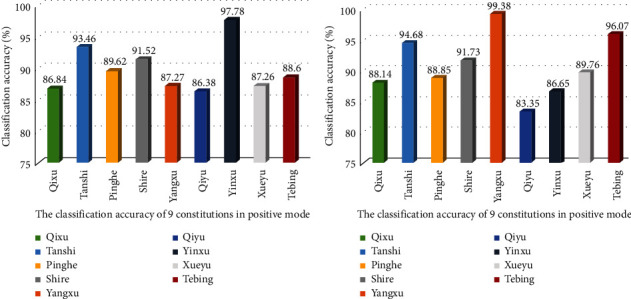
Test results of the constitution discrimination model. (a) Test results under positive mode conditions. (b) Test results under negative mode conditions.

The verification results of 9 constitutions are shown in Figure 6. Under the positive mode, the overall classification accuracy is 89.66%, among which the average classification accuracy of Yinxu constitution is the highest, reaching 97.78%, and the average classification accuracy of Qiyu constitution is the lowest, reaching 86.38%. Under the condition of negative mode, the overall classification accuracy was 90.68%, among which the average classification accuracy of Yangxu constitution was the highest, reaching 99.38%, and the average classification accuracy of Qiyu constitution was the lowest, reaching 83.35%.

## 4. Discussion

According to the test results of the model, the overall classification accuracy rate and the average classification accuracy rate of each constitution are both higher than 80%, indicating that the constructed model can distinguish the 9 constitutions of TCM well, and the classification accuracy rate may be related to the difficulty of distinguishing each constitution. The classification accuracy of Yinxu constitution and Yangxu constitution is high, which may be related to the fact that Yinxu constitution and Yangxu constitution are easily distinguished from other constitutions [27, 28]. The classification accuracy of Qiyu constitution is low in both models, which may be related to the difficulty in distinguishing Qiyu constitution from other constitutions or the number of characteristic *m/z* values of qi depression constitution [29].

The nine constitution discrimination models of traditional Chinese medicine constructed in this study are based on nine simple constitutions and do not consider the mixed constitution. However, people with mixed constitution are not rare in the general population, so the model cannot judge the constitution of people with mixed constitution. The nine-constitution discrimination model database of traditional Chinese medicine constructed by us can continuously expand the data, continue to receive the characteristic information of each constitution, and is expected to distinguish many samples to achieve the purpose of scientifically, objectively, and quickly identifying the nine constitutions of traditional Chinese medicine. However, due to the small sample size used for modeling at present, we need to conduct larger-scale research to further explore the differences in the mechanism of each constitution, provide services for clinical physique identification. [30, 31].

By studying the physiological and pathological reaction states of human body and individual differences, TCM constitution divides people into different constitution types, discusses the relationship between different constitution types and diseases, and then realizes the purpose of preventing and treating diseases by intervening in different constitutions. Tian et al. [32] analyzed and discussed the historical evolution of TCM constitution identification, the guidance of TCM constitution identification for syndrome differentiation and treatment, and the guiding significance of TCM constitution identification for prevention and treatment of COVID-19. They proposed the application of TCM constitution identification theory to analyze the physical characteristics of different populations and carry out early intervention and correction. It may reduce the incidence of COVID-19, and the treatment of COVID-19 should be based on regional and climate differences, combined with the physical characteristics of different individuals, to achieve a combination of syndrome differentiation and body differentiation, to improve the clinical efficacy, to provide theoretical reference for the application of TCM constitution identification in the prevention and treatment of COVID-19. Xie et al. [33] showed that TCM constitution was a risk factor affecting the occurrence of cardiovascular disease in community residents, among which the risk of cardiovascular disease was significantly higher in people with biased constitution, such as qi deficiency, Yin deficiency, blood stasis, and blood deficiency. Hu et al. [34] showed that phlegm-dampness constitution, Yang deficiency constitution, and damp-heat constitution are the main biased constitution types of patients with polycystic ovary syndrome (PCOS), and phlegm-dampness constitution, damp-heat constitution, and blood stasis constitution may be risk factors for the occurrence of polycystic ovary syndrome (PCOS). Yao et al. [35] showed that the physical characteristics of patients with alcoholic liver disease were damp-heat constitution, while those with alcoholic hepatitis tended to be qi stagnation constitution and blood stasis constitution. The constitution of patients with alcoholic cirrhosis is partial to blood stasis constitution and phlegm-dampness constitution.

## 5. Conclusion

In conclusion, these studies show that constitution types are closely related to diseases, and TCM constitution identification is an important method to determine different individual constitution types. Therefore, it is possible to prevent and treat diseases by good constitution identification [36, 37].

TCM constitution identification has played and will continue to play an important role in guiding TCM practitioners to prevent and treat human diseases. In this study, based on metabolomics data, a discriminant model of nine physiques of traditional Chinese medicine was constructed, and new method and idea were proposed. The model database can continuously expand data, continue to accept new feature information of each physique, and is expected to be able to identify many samples to achieve the objective, scientific, and rapid identification of physique. Although this study can provide an objective basis for the identification of physiques in traditional Chinese medicine, more research is still needed to further explore the differences in the mechanisms of various constitutions.

## Figures and Tables

**Table 1:** Standard table of Pinghe constitution and 8 kinds of biased constitution.

Constitution	Scoring conditions	Results
Pinghe	The conversion score of Pinghe constitution scale was ≥60 points, and the conversion score of the other 8 biased constitution scales was <30 points	Yes
	The conversion score of Pinghe constitution scale was ≥60 points, and the conversion score of the other 8 biased constitution scales was <40 points	Probably yes
	Failure to meet the above requirements	No

8 kinds of biased constitution	The conversion score of Pinghe physique scale was ≥60, and the conversion score of one kind of biased physique scale was ≥40	Yes
	The conversion score of Pinghe physique scale was ≥60, and the conversion score of one kind of biased physique scale was 30–39	Probably yes
	The conversion score of Pinghe constitution scale was ≥60 points, and the conversion score of the other 8 biased constitution scale was <30 points	No

**Table 2:** Comparison of general data of volunteers with 9 kinds of constitution in TCM.

Group	The number of cases	Gender		Age (years)
		Male	Female	
Qixu	49	12	37	26.41 ± 4.11
Tanshi	49	16	33	28.98 ± 4.41
Pinghe	49	12	37	26.96 ± 4.37
Shire	49	16	33	26.73 ± 2.94
Yangxu	49	10	39	27.57 ± 4.33
Qiyu	49	13	36	26.69 ± 2.59
Yinxu	49	14	35	26.31 ± 4.75
Xueyu	49	15	34	26.67 ± 2.14
Tebing	49	11	38	28.51 ± 4.21

**Table 3:** *m/z* values of eight biased constitutions compared with Pinghe constitutions.

Constitution	Differential *m/z* values in positive mode	Differential *m/z* values in negative mode
Qixu constitution	261	580
Tanshi constitution	300	588
Shire constitution	726	531
Yangxu constitution	248	234
Yinxu constitution	267	420
Qiyu constitution	36	43
Xueyu constitution	285	323
Tebing constitution	876	410

**Table 4:** The number of *m/z* values unique to the eight biased constitutions.

Constitution	Unique *m/z* values in positive mode	Unique *m/z* values in negative mode
Qixu constitution	5	26
Tanshi constitution	18	37
Shire constitution	104	49
Yangxu constitution	52	16
Yinxu constitution	11	32
Qiyu constitution	0	2
Xueyu constitution	23	161
Tebing constitution	209	64

## Data Availability

The original contributions presented in the study are included in the Supplementary Materials; further inquiries can be directed to the corresponding author.
